# Cavernous Sinus Dural Arteriovenous Fistula in a Patient with Thyroid-Associated Ophthalmopathy: Complete Resolution after Medical Treatment

**DOI:** 10.3390/brainsci12010045

**Published:** 2021-12-29

**Authors:** Nicola Cavasin, Fabio Presotto, Matteo Bellamio, Enrico Cagliari

**Affiliations:** 1 Department of Neuroradiology, Ospedale dell’Angelo, Via Paccagnella 11, 30174 Mestre, Italy; enrico.cagliari@aulss3.veneto.it; 2 Department of Internal Medicine, Ospedale dell’Angelo, Via Paccagnella 11, 30174 Mestre, Italy; fabio.presotto@aulss3.veneto.it; 3 Department of Neurology, Ospedale dell’Angelo, Via Paccagnella 11, 30174 Mestre, Italy; matteo.bellamio@aulss3.veneto.it

**Keywords:** thyroid-associated ophthalmopathy, cavernous sinus dural fistula, endovascular embolization

## Abstract

Thyroid-associated ophthalmopathy (TAO) is a well-known and frequent epiphenomenon of a hyperthyroid autoimmune disease that can present with proptosis, strabismus, and diplopia. Ophthalmopathy can occur in the absence of overt Graves’ disease, even in euthyroid patients. Cavernous sinus dural fistulas (CS-DAVF) are abnormal communications between the cavernous sinus (CS) and dural branches from internal carotid or external carotid arteries. They can often present with ocular symptoms that can mimic a thyroid-associated ophthalmopathy. CS-DAVF are usually successfully treated with an endovascular embolization that can be pursued both through a transvenous or transarterial approach. TAO and CS-DAVF can coexist especially when the ocular symptoms are unilateral. In those cases, an endovascular embolization is usually curative, but sometimes the procedure can fail. Our hypothesis is that some cases of CS-DAVF may be of secondary nature (i.e., caused by compression of the venous outlet by the hypertrophic ocular muscles); therefore, treating the ocular disease with medical therapy may solve the vascular problem as well. We present a case of a CS-DAVF in a patient with TAO successfully treated with sole medical therapy after the failure of a first-line endovascular treatment.

## 1. Introduction

Thyroid-associated ophthalmopathy (TAO) is an autoimmune disorder representing a crucial extrathyroidal manifestation of Graves’ disease, but it may occur in patients without current or prior hyperthyroidism (euthyroid or ophthalmic Graves’ disease) or in patients with chronic autoimmune (Hashimoto’s) thyroiditis [1,2]. It is thought to be related to immune-mediated activation of fibroblast and the accumulation of glycosaminoglycans and water in the extraocular muscles. Additionally, fat, connective tissue, and muscle expansion within the bony orbit can result in proptosis (exophthalmos), strabismus (eye misalignment), and diplopia (double vision). In 10% of the cases, ophthalmopathy can occur in the absence of overt Graves’ disease and can seldom be found in euthyroid patients [3,4]. Cavernous sinus dural fistulas (CS-DAVF) are abnormal communications between the cavernous sinus (CS) and dural branches from internal carotid or external carotid arteries that are thought to be acquired [5]. An extensive anastomotic network of vessel physiologically exists on the surface and within the cavernous sinus, and these vessels can sometimes overdevelop under pathological circumstances. CS-DAVFs are generally acquired, and several conditions can act as cofactors in its development such as pregnancy, systemic hypertension, atherosclerotic vascular disease, fibromuscular dysplasia, and Ehlers–Danlos type IV; there is a female predominance, particularly in the postmenopausal age. Etiopathogenesis is not clear. The most favored theory indicates the CS-DAVF as a response to venous thrombosis that can occur spontaneously in one or many of the multiple channels that constitute the cavernous sinus in an attempt to rebuild a collateral pathway [6]. Orbital venous congestion and even thrombosis of the orbital venous outflow can be a complication of thyroid-associated ophthalmopathy that can occur either from crowding at the orbital apex or from impingement of the superior rectus muscle on the superior ophthalmic vein [7]. We hypothesize that the same pathomechanism leading to superior ophthalmic vein stenosis or occlusion in patients affected by thyroid-associated disease can sometimes be associated with CS-DAVF’s formation. There are scarce reports about the association of CS-DAVF and TAO, and CS-DAVFs can often present with ocular symptoms that can mimic a TAO. CS-DAVF are usually successfully treated with an endovascular embolization that can be pursued both through a transvenous or transarterial approach. We present a case of a CS-DAVF in a patient with TAO that regressed with medical therapy after the failure of a first-line endovascular treatment.

## 2. Case Report

**Figure 1:**
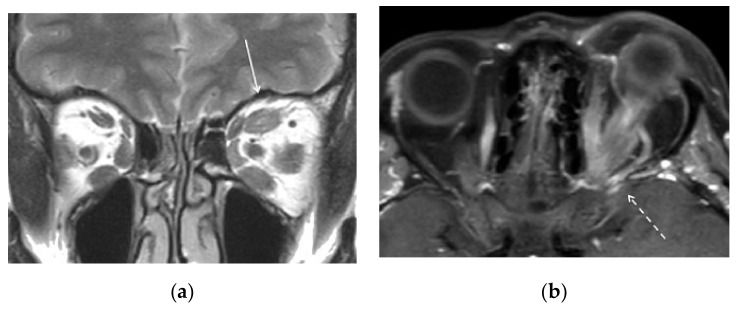
(**a**) MR, T2-weighted image, coronal plane, confirming a bilateral global hypertrophy of the extrinsic muscles, more pronounced to the left superior rectus (white arrow). (**b**) MR, T1-weighted fat-sat postgadolinium image, axial, showing a slightly enlarged left superior ophthalmic vein (dashed arrow), partially pinched by the superior rectus muscle.

A 68-year-old female was admitted to our emergency department referring in the last two months a left retro-orbital pain associated with lacrimation and visual disturbance. Objectively, she presented redness and edema of the conjunctiva with spontaneous retrobulbar pain and pain on up or down gaze (Clinical Activity Score > 3/7). She had subjective intermittent diplopia due to eye motility impairment without signs of optic nerve involvement, such as visual acuity loss. She had two sisters affected by thyroid disease, and her personal history was significant for past smoking, hypercholesterolemia, and inflammatory bowel disease. She had also previously been diagnosed with euthyroid Hashimoto’s thyroiditis with a slight increase in antibodies to thyroid peroxidase (TPOAbs) and normal levels of antibodies to the TSH receptor (TRAbs). General and neurological examination did not show any other significant finding. The unilateral involvement, the euthyroid state, and the negligible TPOAbs and TRAbs titolation were not deemed to be related to the underlying subclinical thyroid disease and led to further investigations in order to rule out potential secondary causes of unilateral retro-orbital disease (i.e., brain or retro-orbital tumors, vascular diseases, or malformations). Orbital MR scans (Philips Ingenia CX 3T scanner. T1 [TR 539.4, TE 14] and T2 [TR 2500, TE 90] weighted sequences, with slice thickness of 3 mm, on the coronal plan, and T1 weighted [TR 446.2, TE 8.0], with slice thickness of 2.5 mm, on the axial plan. All sequences were acquired with the chemical shift-based water–fat separation Dixon method) confirmed a left exophthalmos and showed global left extrinsic ocular muscles increase in size without significant hypertrophic superior or inferior ophthalmic vein (Figure 1a,b).

**Figure 2:**
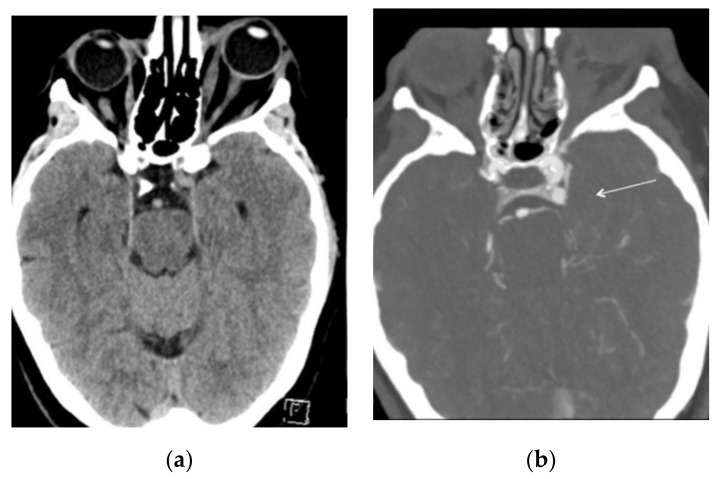
(**a**) CT scan on admission showing left exophthalmos with hypertrophic extrinsic ocular muscles. (**b**) CT angio on the same day demonstrating a small venous pouch on the posterior aspect of the left cavernous sinus (white arrow).

Intracranial CT angiography (Siemens Somatom Definition Flash, 80–100 kV, 151–159 mAs, 0.6 mm thick slices and 512 × 512 matrix) demonstrated an anomalous small venous pouch on the posterior aspect of the left cavernous sinus (Figure 2a,b).

**Figure 3:**
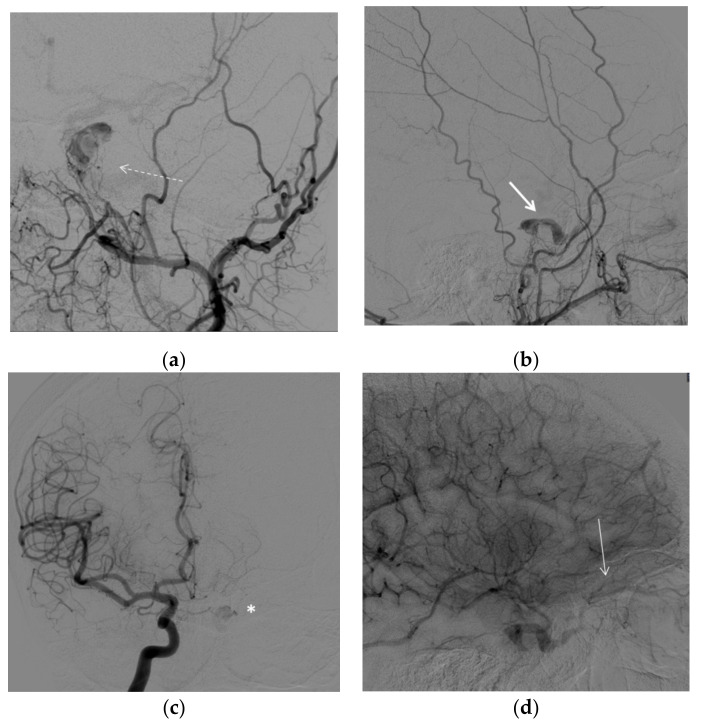
DSA. (**a**) Left external carotid artery, anterior oblique view showing several, tiny branches from the internal maxillary artery feeding the CS-DAVF (dashed arrow). (**b**) Left external carotid artery, lateral view, demonstrating early opacification of the left cavernous sinus (arrow) due to the dural fistula. (**c**) Right internal carotid artery, antero-posterior view. Small branches from the carotid syphon converge to the contralateral cavernous sinus (asterisk) through the intercavernous plexus. (**d**) Right internal carotid artery, latero-lateral view, late phase. The retrogradely opacified left superior ophthalmic vein is proximally enlarged and slightly pinched where it crosses the superior rectus muscle (arrow).

A vascular malformation was then suspected, and a digital subtraction angiography (DSA) was then performed. On angiography, a CS-DAVF was found on the left side (Figure 3a–c), with arterial feeders from dural branches of the ipsilateral maxillary artery and the contralateral carotid syphon and a slow drainage through the left superior ophthalmic vein. However, the draining vein was unexpectedly small (Figure 3d).

Due to the annoying symptoms and the potential complications of a long-standing CS-DAVF to the eye function, an endovascular treatment was recommended. CS-DAVF’s usual pattern is that of a malformation with multiple tiny arterial feeders that converge on the dural layer of the CS. The venous outflow of the malformation is toward the less resistance venous route: it can be through the orbit via the ophthalmic veins (as in the present case) or even toward the intracranial venous circulation. In order to achieve the complete cure of the disease, the point of fistula has to be occluded, and in these kinds of malformations, the best way to achieve it is disconnecting the arterio-venous shunts at the venous side (i.e., the CS) through endovascular embolization.

**Figure 4:**
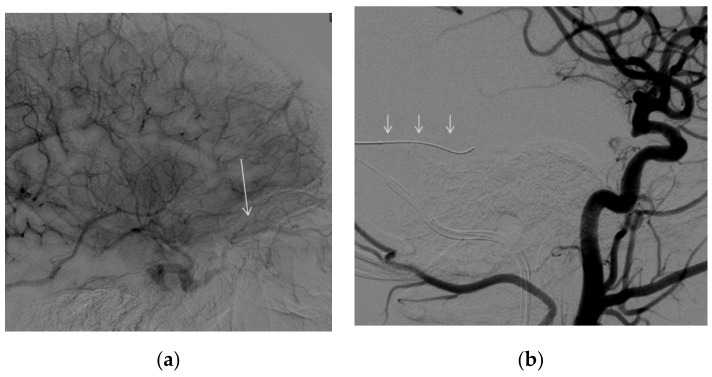
DSA. Embolization procedure. Left common carotid artery, lateral view. (**a**) Left inferior (arrow) and (**b**) left superior petrosal sinus (small arrows) catheterization. No access to the venous pouch of the fistula could be found.

A transvenous approach was then attempted. The procedure was performed, under general anesthesia, through a venous and arterial femoral access. A full dose of 5.000 UI of intravenous unfractionated heparin (sodium heparin) was administered at the beginning of the procedure. On the venous side, we used a guiding catheter with the distal tip at the left jugular bulb. On the arterial side, we used a diagnostic catheter with the distal tip in the left maxillary artery for diagnostic purpose. From the venous side, we attempted with microcatheter and microguidewires to access the left inferior or left superior petrosal sinus to retrogradely access the diseased portion of the CS and occlude it with coils with a technique that has previously been described and is generally associated with high rates of success [8,9]. Nevertheless, despite several attempts, we could not negotiate the venous pouch (Figure 4a,b).

The CS is not a single cavity; it is a dural pouch that lies on both sides of the sella working as a venous collector for both intracranial and extracranial circulation, and it contains multiple septa, thus creating different small cavities. Therefore, from an endovascular point of view, it can happen to access one “subcavity” of the sinus that is completely separated from the others. Transarterial embolization is quite often a valuable alternative in CS-DAVF treatment, but in this case, the arterial feeders were tiny “en passage” vessels that would have probably been embolized and occluded before reaching the point of fistula; moreover, the occlusion of these meningeal arteries could cause cranial nerve deficits [6]. For these reasons, we decided to abort the procedure. After the failure of the endovascular procedure, an alternative treatment should have been proposed to the patient. Assuming that the extrinsic muscle hypertrophy might be the cause of the venous congestion by external compression on the superior ophthalmic vein, we tried a different approach: we speculated that the CS-DAVF might have been a consequence of the extrinsic ocular muscles hypertrophy. Based on this assumption, steroid treatment regimen was started for moderate-to-severe ophthalmopathy according to the EUGOGO Consensus statement [10,11], which is to administrate weekly intravenous methylprednisolone at high doses in 12 weeks. The standard protocol suggests an i.v. bolus of 500 mg/week for 6 weeks then 250 mg/week for 6 more weeks, but in this case of active TAO, we employed higher cumulative doses, i.e., 500 mg/i.v./week for 12 weeks (cumulative dose of 6.0 g of methylprednisolone), continuing with a tapering oral administration of prednisone (initial dose of 25 mg) in the following month. The patient was discharged with a schedule of a 3 and 6 month CT angio and concomitant clinical examinations.

**Figure 5:**
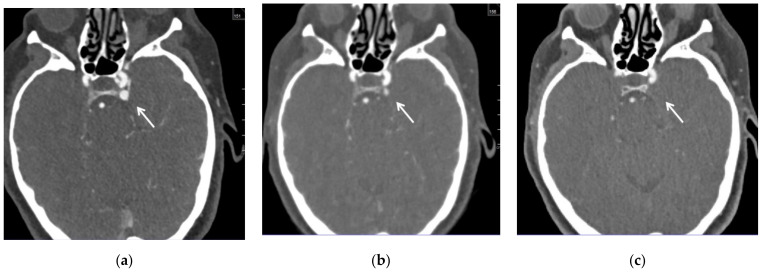
CT angio on admission (**a**) and at 3 (**b**) and 6 (**c**) month follow-up. The venous pouch at the posterior aspect of the left cavernous sinus (white arrow) progressively decreased in size and disappeared.

**Figure 6:**
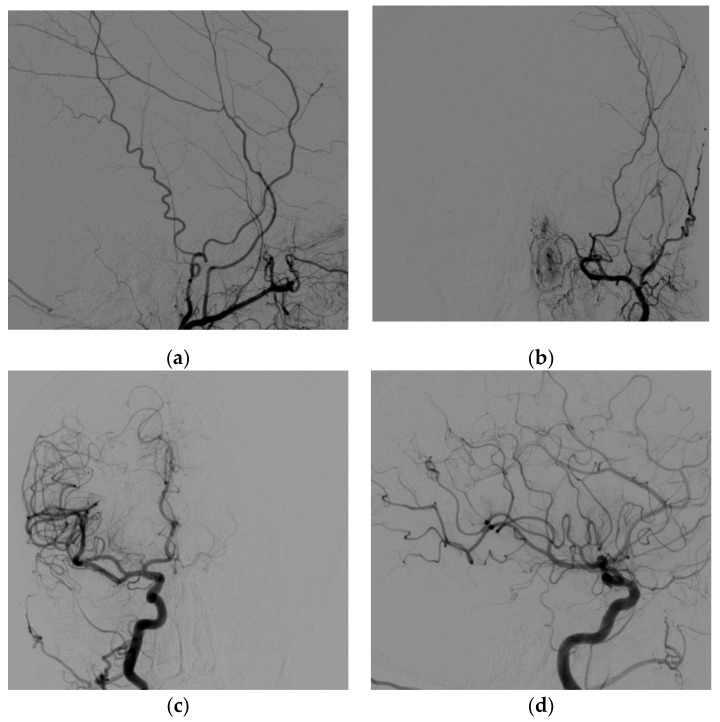
DSA at 7 months, left external carotid artery, lateral (**a**) and antero-posterior (**b**) views. right internal carotid artery antero-posterior (**c**) and lateral (**d**) view. The DSA shows complete resolution of the dural fistula.

The follow-up CT angio examinations showed that the venous pouch of the CS-DAVF progressively decreased in size (Figure 5a–c), and at the final DSA after the end of the cycle of corticosteroid therapy (performed 7 months after discharge), it was completely occluded (Figure 6a–d). From the clinical point of view, the proptosis regressed, and diplopia and retroorbital pain disappeared; a slight conjunctival hyperemia remains but at three years, the patient is still free of symptoms.

## 3. Discussion

One-sided retro-orbital pain associated with ipsilateral lacrimation, conjunctival injection, chemosis, proptosis, and diplopia could be attributed to a primary form of trigeminal autonomic cephalalgias (TACs) such as cluster headache, especially at its first presentations [12]. The duration of the pain, the worsening of symptoms, and the physical evolution of ocular alterations led us to further investigations to exclude secondary and potentially dangerous diseases such as tumors, inflammation, infections, or vascular abnormalities (i.e., arterovenous malformations, cerebral venous thrombosis, or intracranial dural fistulas, as in this case) [13,14]. Intracranial dural fistulas are acquired, abnormal connections between dural arteries from the internal and external carotid or vertebral arteries and the dural sinuses or their closest venous collectors; they can also receive contributions from pial branches. The clinical features are extremely variable depending on the location and the subtype of the fistula and on the angioarchitecture of the disease itself. A CS-DAVF is a well-known subtype of dural fistulas in which dural feeders from the internal and external carotid arteries connect with the CS. They account for 10–15% of all cerebral vascular malformations [15]. They develop spontaneously and differentiate from other vascular diseases affecting this region, such as the so-called direct carotico-cavernous fistulas that are high-flow, single-hole communications between the carotid syphon and the CS that can be caused by the rupture of a carotid syphon aneurysm or a direct trauma (i.e., skull base fractures). The Barrow classification categorizes these vascular conditions into four subtypes according to their anatomical features provided by angiographical studies [5]: Type A fistulas are direct shunts between the internal carotid artery and the CS, usually post-traumatic or due to the rupture of a carotid syphon aneurysm, “indirect” fistulas are dural arteriovenous fistulas of the CS fed by dural branches of the internal carotid artery (Type B), of the external carotid artery (Type C) or both (Type D). Our case featured the pattern of a Type D fistula. The CS is an epidural plexus located between the periosteum and the dura mater on both sides of the sella. It is a crucial venous hub as it drains the face-orbit region as well as the brain, and it can function as a rerouting pathway in case of obstruction or diseases affecting the normal venous outlets; anteriorly, it receives the superior and inferior (or common venous confluent) ophthalmic veins [9]. In CS-DAVF, several abnormal small arterial feeders converge to the CS plexus so that the normal flow within the plexus is increased and a venous hypertension is created. This determines flow reversal in one or many of the veins that anastomose with the CS itself. The superior ophthalmic vein is the most common drainage in CS-DAVFs; in this condition, it often becomes enlarged and engorged and determines, mainly by mass effect on extrinsic ocular muscles and the optic nerve, the clinical features of this disease. Patients affected by a CS-DAVF can present with eye pain, proptosis, diplopia, conjunctival injection, chemosis, and loss of vision [5]. The CS communicates with many other venous collectors; on the lateral side, it receives the cortical veins (i.e., the sylvian veins) through the sphenoparietal sinus [15]. In cases of CS-DAVF with drainage into cortical veins, the most feared clinical issue can be cerebral hemorrhage. Indications for CS-DAVF treatment are severe or progressive symptoms and/or cortical venous reflux (for the hemorrhagic risk). Endovascular treatment is the first-line option through transvenous coil obliteration of the diseased portion of the sinus or, more precisely, of the fistulous point. In a review from a Japanese group [16], this treatment showed great rates of success with complete disappearance of the fistula in 72.5% of the series (median), immediately after the procedure. This rate increases up to 100% (in the series reporting long-term follow-ups). The reason is that even in those cases with a partial shunt occlusion immediately after the procedure the disappearance of the fistula progresses for the thrombogenic effect of the coils and not only for the direct packing of the sinus [16]. The embolization of the several narrow and tortuous arterial branches that feed the fistula is usually not only time consuming and very often technically demanding but can also be a treatment failure because it is easy to miss the arterio-venous shunt because the small diameter of the arterial feeders does not allow for the deep penetration of our embolic materials. Several venous routes are available for endovascular access to the CS but not all are straightforward, and in this case, the venous outlets of the fistula were not large enough and easily accessible. A surgical exposure of the SOV or the CS is possible [8] but the risk-to-benefit ratio was deemed unfavorable. The association of TAO and CS-DAVF has seldom been reported. Lore et al. [17] presented the case of a woman affected by Graves’ disease, with previous hyperthyroid state, treated for 10 years with methimazole then with ^131^I. She was admitted for a bilateral progressive ophthalmopathy, in a state of subclinical hyperthyroidism, initially treated with steroids. She presented only asymmetric improvement of the ocular symptoms, and this feature led clinicians to perform further investigations that demonstrated the presence of a CS-DAVF. She was successfully treated with transvenous embolization with immediate clinical improvement. Celik and colleagues [18] reported one more case: a woman with known Graves’ disease with progressive ophthalmopathy. She was initially treated with glucocorticoids but partially responded with residual right exophthalmos. A subsequent angiography showed the presence of a right CS-DAVF with bilateral superior ophthalmic vein drainage, more pronounced on the right side. An endovascular embolization of the right CS was performed, leaving untouched the small left-sided venous drainage, with regression of the symptoms and radiological improvement. In the two reported cases, the patients have been initially treated with glucocorticoids, and only after partial failure of the pharmacological approach and asymmetric persistence of ocular signs and symptoms was a dural fistula suspected. TAO generally is associated with autoimmune hyperthyroidism, but in 5–10% of the cases, it can occur with hypothyroidism or normal thyroid function [1,2]. In these cases, the diagnosis of TAO is based on the presence of one or more autoimmune thyroid-associated autoantibodies. Antibodies anti-thyroid peroxidase (TPO-Ab) are generally associated with main chronic autoimmune thyroid disorders (Hashimoto’s thyroiditis, Graves’ disease), while autoantibodies against the TSH receptor (TRAb) are specific and pathogenic for Graves’ disease or mixed thyroid autoimmune thyroid disorders (Hashitoxicosis). In the presence of ocular symptoms and the clear absence of thyroid disfunction or antibodies, such as in our case, a different etiology must be taken into account, and further imaging workout has to be planned (brain CT or MR) to exclude other causes of ophthalmopathy (CS-DAVFs, meningiomas, lymphomas, cellulitis, pseudotumor cerebri, metastatic tumors, and sarcoidosis). The possible causes for negative TRAbs are technical issues, such as the use of an assay lacking adequate sensitivity to detect low level of antibodies, especially in patients with mild disease, or the presence of intrathyroidal, noncirculating antibodies. Furthermore, other factors are involved in the pathogenesis of TAO, such as the insulin-like growth factor receptor (IGF1R), which can be expressed on the surface of orbital fibroblast cells and can be one of the target autoantigens in TAO [19]. Patients with clinical features that fit with the diagnosis of TAO should receive a thorough evaluation with imaging studies even if the laboratory tests are negative: the full expression of the thyroid disease can develop years after the orbital signs. The relative asymmetrical involvement of the extrinsic ocular muscles in our case and the presence of a CS-DAVF led us to think that the ocular disease can be primarily a consequence of the vascular pathology, whereas its complete disappearance with the reduction of the edema to the muscles after medical therapy is in favor of a secondary nature of the fistula. A direct effect of the extrinsic ocular muscle disease on the orbital veins in TAO was postulated long ago [7,19] and has extensively been demonstrated. Enlargement and stasis and even thrombosis of the superior ophthalmic vein can develop mainly from apical crowding or enlargement of the superior rectus muscle or even muscle inflammation, which involves the vascular network leading finally to a periphlebitis. Venous congestion or venous thrombosis is one of the primary causes inducing dural fistulas development.

We think that the prevalence of CS-DAVF in patients with TAO is underestimated. Many cases that did not undergo further imaging studies for an asymmetrical but bilateral ophthalmopathy could have been affected by a concomitant dural fistula that was missed simply because the clinical signs and the symptoms improved because they were successfully treated with glucocorticoids. These TAO-associated CS-DAVF can therefore be considered as having a benign course, and a pharmacological first-line treatment can be justified. Endovascular treatment with transvenous or transarterial embolization can be reserved for clinically progressive cases or sight-threatening conditions or in cases of failure of medical therapy.

## 4. Limitations

We described a case of complete and stable remission of a CS-DAVF and symptoms in a patient with concomitant TAO. We know that the two disorders can variably improve spontaneously. The ocular symptoms of TAO can improve and regress without specific treatment, and spontaneous regression of CS-DAVF is a well-known event. It has been reported to occur in 10–73% of the cases, especially in cases of low-flow and low-pressure shunts within a time-window of 12 months or longer, but the angiographic confirmation of the disappearance is seldom pursued. It can be argued that in our case, complete resolution of the symptoms and healing of the CS-DAVF could have been obtained even without glucocorticoid therapy, but the chronological connection between the onset of symptoms, the treatment, and the rapid resolution of the symptoms and of the fistula are clear, and from an anatomical point of view, the clear reduction in size of the extraocular muscles has reduced the mass effect on the superior ophthalmic vein with a restoration of the normal venous outflow.

## 5. Conclusions

To our knowledge, there are to date no publications that report the complete disappearance of a dural fistula associated with sole medical therapy. This is a single case, and further data are needed to confirm a potential correlation, but it may suggest a secondary nature of the dural fistula in patients with TAO.
